# Power Doppler ultrasonography for positional graft compression after femoral artery reconstruction: A case report

**DOI:** 10.1097/MD.0000000000050892

**Published:** 2026-09-25

**Authors:** Taisuke Furuta, Mitsuo Ochi, Tomohiko Sakuda, Yuko Nakashima, Rikuo Shinomiya, Nobuo Adachi

**Affiliations:** a Department of Orthopaedic Surgery, Graduate School of Biomedical and Health Sciences, Hiroshima University, Hiroshima, Hiroshima, Japan; b Hiroshima University, Higashi-Hiroshima, Hiroshima, Japan; c Department of Medical Transdisciplinary Education, Hiroshima University, Hiroshima, Hiroshima, Japan; d Department of Musculoskeletal Traumatology and Reconstructive Surgery, Graduate School of Biomedical and Health Sciences, Hiroshima University, Hiroshima, Hiroshima, Japan.

**Keywords:** ankle–brachial index, great saphenous vein, power Doppler ultrasound, radiation-induced vasculopathy, soft tissue sarcoma, vascular reconstruction

## Abstract

**Rationale:**

Vascular reconstruction with autologous vein grafts in musculoskeletal tumors poses biomechanical challenges.

**Patient concerns:**

A 35-year-old man developed pain and numbness during deep knee flexion 3.5 years after femoral artery reconstruction using the great saphenous vein.

**Diagnoses:**

Power Doppler ultrasonography revealed positional vascular compromise in the reconstructed graft.

**Interventions:**

Conservative management with activity modification.

**Outcomes:**

Symptoms improved with avoidance of deep flexion; no further progression was noted.

**Lessons:**

Dynamic Doppler ultrasonography is a simple, noninvasive method for diagnosing mechanical graft compression after vascular reconstruction.

## 1. Introduction

Vascular involvement in malignant musculoskeletal tumors often necessitates resection and subsequent reconstruction of major vessels. While prosthetic grafts are available, autologous vein grafts – particularly the great saphenous vein – are generally preferred because of their superior biocompatibility and lower infection risk, especially in regions near joints or contaminated fields.^[1,2]^ Nevertheless, even autologous vein grafts may not fully match the elasticity and tensile strength of native arteries, potentially predisposing them to mechanical stress‐related complications.^[3]^

Postoperative complaints of pain or paresthesia are not uncommon, but identifying the precise etiology can be challenging given the anatomical complexity and multimodal treatment often involving chemotherapy and radiotherapy. Vascular and neurologic sequelae of radiation therapy, including radiation‐induced vasculopathy and neuropathy, are well‐recognized late complications in cancer survivors.^[4–8]^

In this report, we describe a rare case of delayed‐onset numbness and pain during deep knee flexion after femoral arterial and venous reconstruction for soft tissue sarcoma. Using power Doppler ultrasonography, we were able to demonstrate dynamic vascular compression of the grafted vessel, providing a simple diagnostic explanation and guiding patient management.

This case report has been reported in line with the Surgical CAse REport 2025 criteria.^[9]^

## 2. Case presentation

A 35-year-old male was referred to our institution for evaluation of a progressively enlarging soft tissue mass located on the posterior aspect of his right thigh. The patient had no significant past medical or surgical history. He did not take any regular medications and had no known drug allergies. There was no family history of malignancy. He did not smoke or consume alcohol. Magnetic resonance imaging (MRI) revealed a 15-cm heterogeneous lesion compressing the popliteal artery, demonstrating low signal intensity on T1-weighted images, a mixture of low- and high-signal intensity on T2-weighted images, and heterogeneous enhancement on gadolinium-enhanced, fat-suppressed T1-weighted sequences (Fig. 1). An incisional biopsy confirmed the diagnosis of myxoid small round cell liposarcoma, classified as stage III (T2bN0M0, histological grade 3) according to the Union for International Cancer Control Tumor, Node, Metastasis classification.

**Figure 1.**
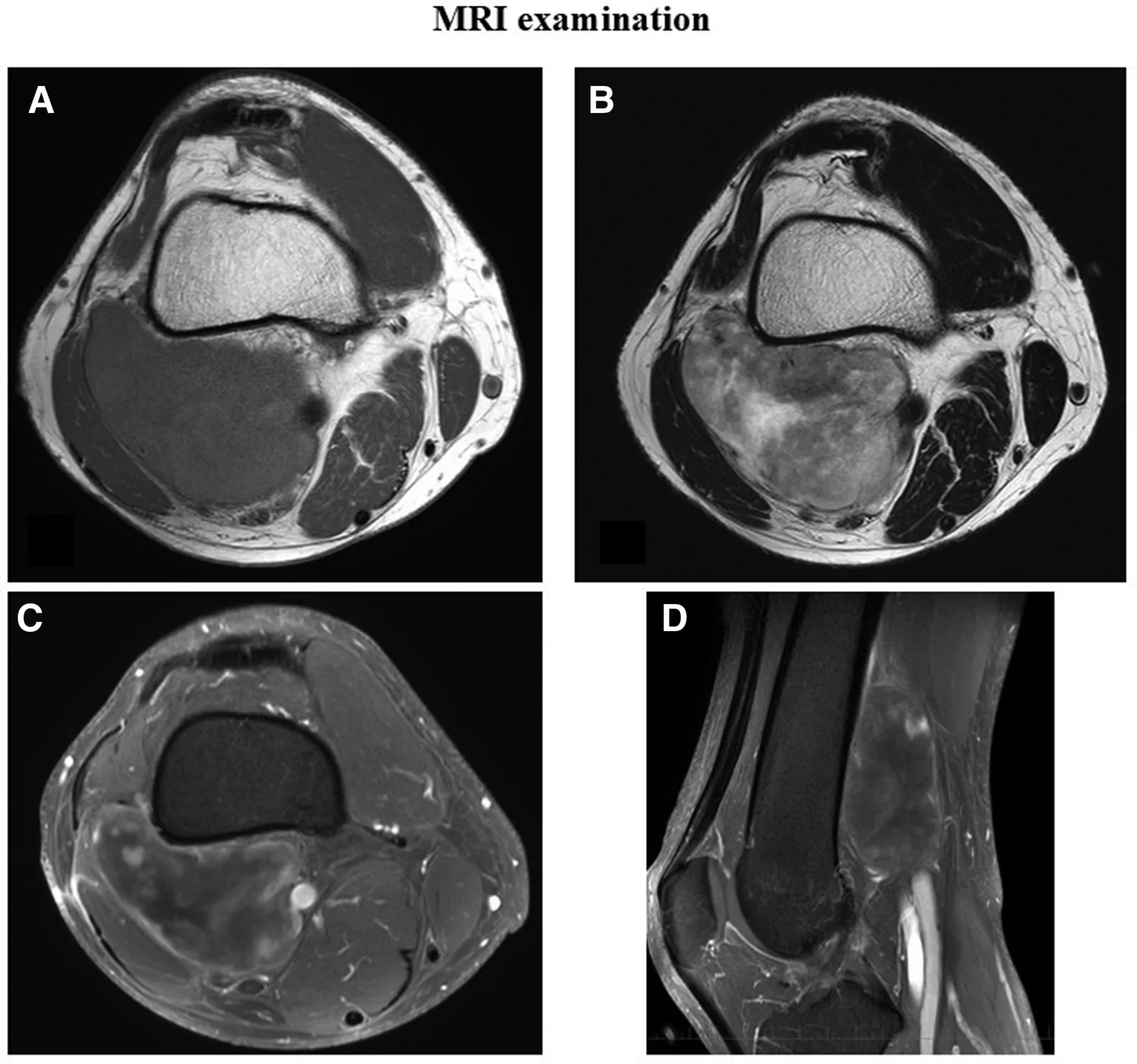
MRI findings. (A) Axial T1-weighted image of the right thigh. (B) Axial T2-weighted image of the right thigh. (C) Axial gadolinium-enhanced fat-suppressed T1-weighted image of the right thigh. (D) Sagittal gadolinium-enhanced fat-suppressed T1-weighted image of the right thigh. (A–D) A tumor approximately 15 cm in size is located on the posterior aspect of the right femur, adjacent to the femoral artery and sciatic nerve. MRI = magnetic resonance imaging.

Given the tumor’s intimate proximity to the femoral artery and sciatic nerve, wide resection with functional preservation was deemed challenging. Neoadjuvant chemotherapy (two cycles of ifosfamide plus adriamycin) was administered. Posttreatment positron emission tomography-computed tomography demonstrated a decrease in SUVmax from 5.0 to 2.1. Based on this favorable response, the surgical plan included extensive tumor excision, partial femoral resection, marginal resection of the sciatic nerve, and vascular reconstruction of the femoral artery and vein.

Although artificial grafts were initially considered, an autologous saphenous vein graft harvested from the contralateral limb was selected to minimize infection risk and ensure flexibility near the knee joint. The affected femur was resected where it abutted the tumor and stabilized with a locking plate. The sciatic nerve was preserved, and vascular continuity was restored using the vein graft (Fig. 2).

**Figure 2.**
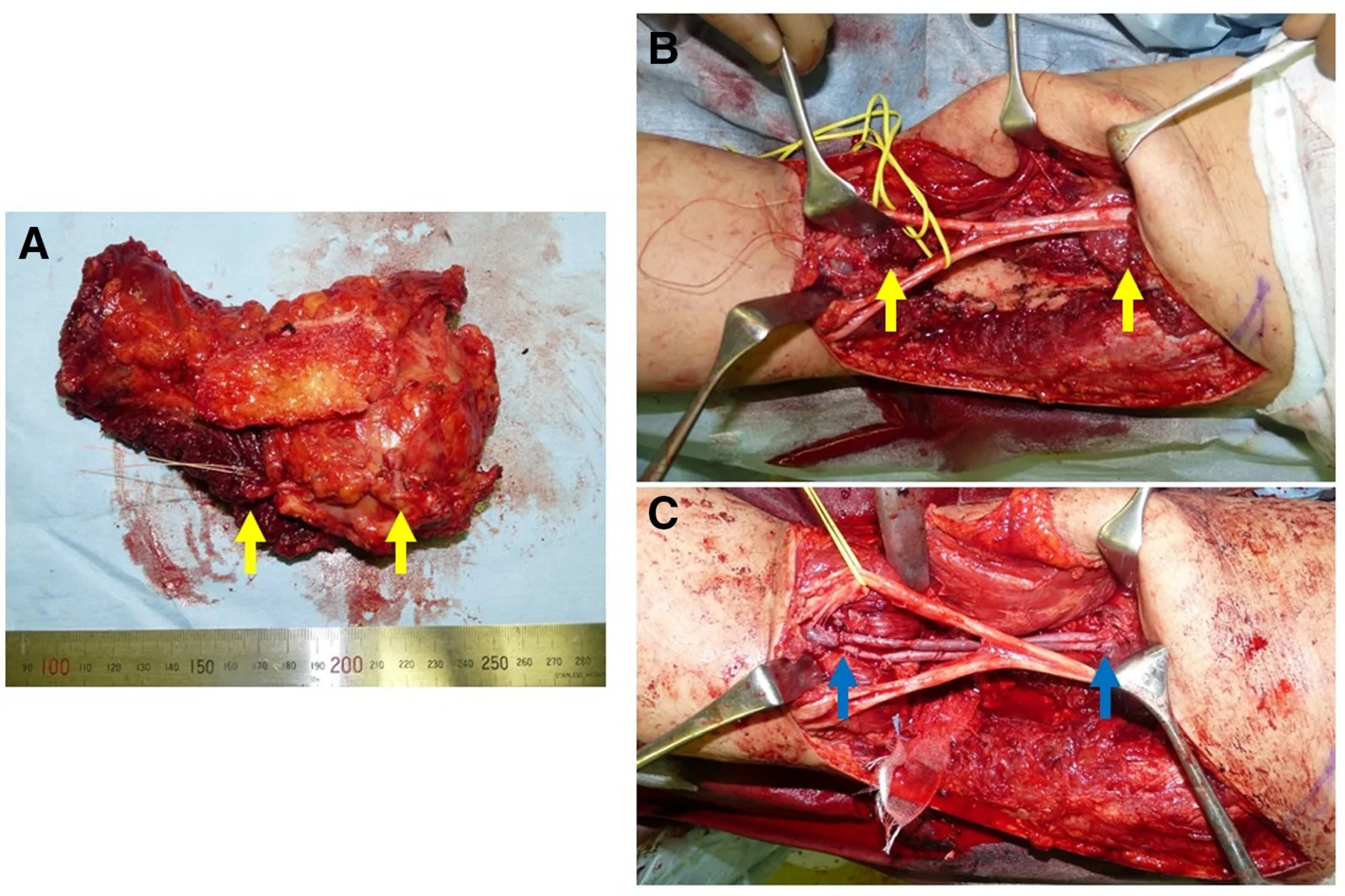
Intraoperative photographs. (A) Resected specimen. (B) Post-tumor resection of the posterior aspect of the right thigh. (C) After revascularization of the femoral artery and femoral vein by the autologous vein. (A) and (B) The yellow arrows indicate the ligation sites of the femoral artery and femoral vein. (C) The blue arrows indicate the sutures of the reconstructed vessel.

Postoperative recovery was uneventful. Adjuvant chemotherapy and radiotherapy (total 60 Gy) were administered. Imaging follow-up (positron emission tomography-computed tomography, MRI) demonstrated no recurrence or metastasis for 3 years postoperatively. The patient achieved a knee range of motion of 0° to 132° without significant complaints.

However, 3.5 years after surgery, the patient reported developing numbness and pain in the lower leg after approximately 10 minutes of sustained knee flexion at around 100°. Notably, as the designated successor to a traditional tea ceremony school, he placed high cultural and personal value on the ability to kneel (seiza position) and was highly motivated to understand the cause of his symptoms.

Neurological examination and lumbar spine MRI excluded a spinal cause. The ankle–brachial index was normal, making peripheral arterial occlusive disease unlikely.

To investigate possible position-dependent vascular compromise, power Doppler ultrasonography was performed using a SNiBLE ultrasound system (Konica Minolta) equipped with a 4 to 18 MHz linear-array transducer. Pulse repetition frequency was adjusted between 800 and 6500 Hz to minimize motion artifacts and optimize flow detection. Blood flow in the dorsalis pedis artery was evaluated in both extension and flexion on the affected and contralateral limbs (Figs. 3 and 4). The reconstructed limb showed complete loss of flow during deep flexion with restoration upon extension, whereas the contralateral side demonstrated only minor flow reduction. Infrared thermography revealed no significant surface temperature differences. These findings supported a diagnosis of positional vascular compression of the graft.

**Figure 3.**
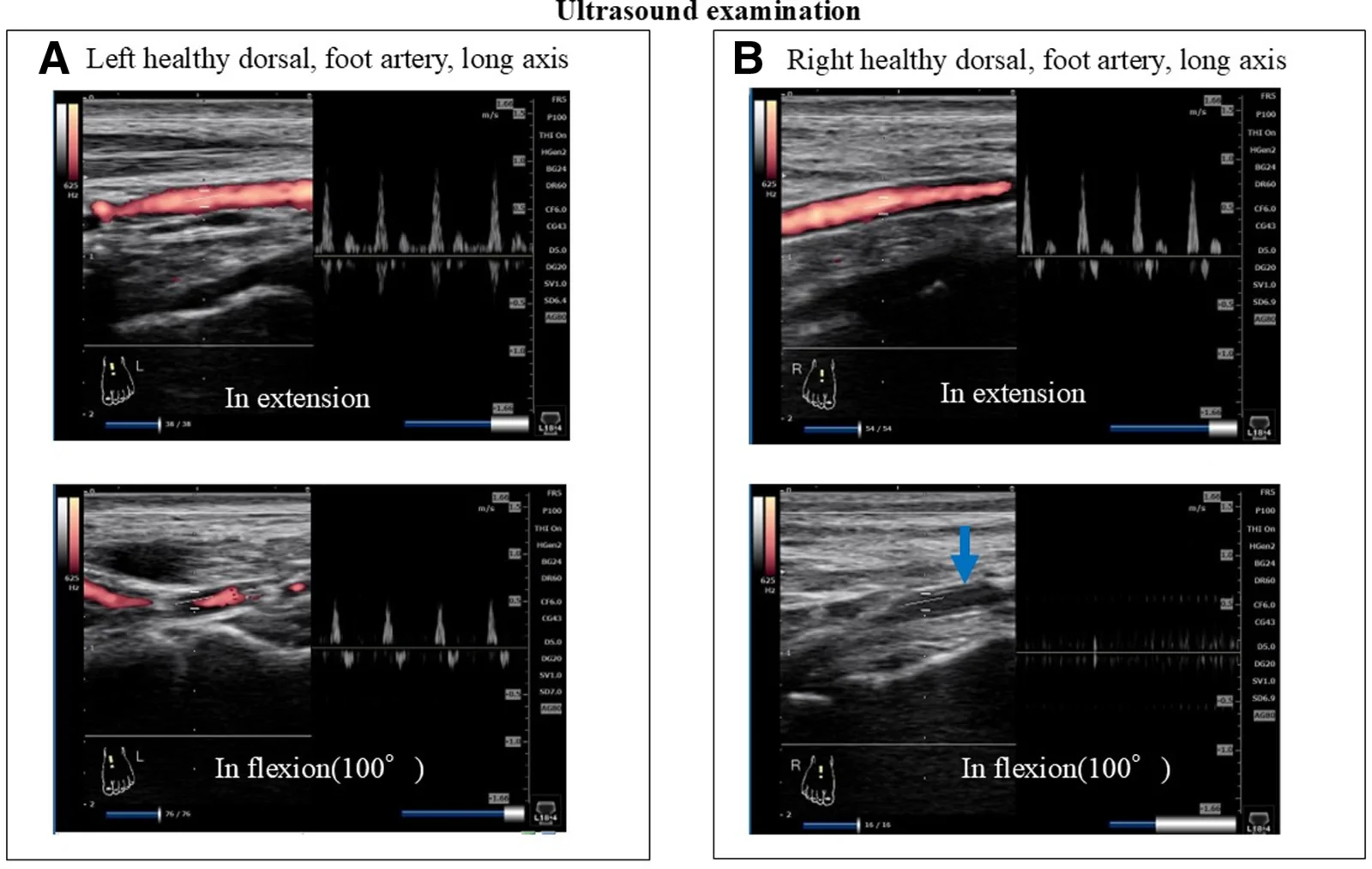
Power Doppler ultrasonography of the dorsalis pedis artery in the long-axis view. (A) Unaffected left limb. Blood flow is maintained during knee extension and is reduced but remains detectable during knee flexion at 100°. (B) Affected right limb. Blood flow is visible during knee extension but disappears during knee flexion at 100°.

**Figure 4.**
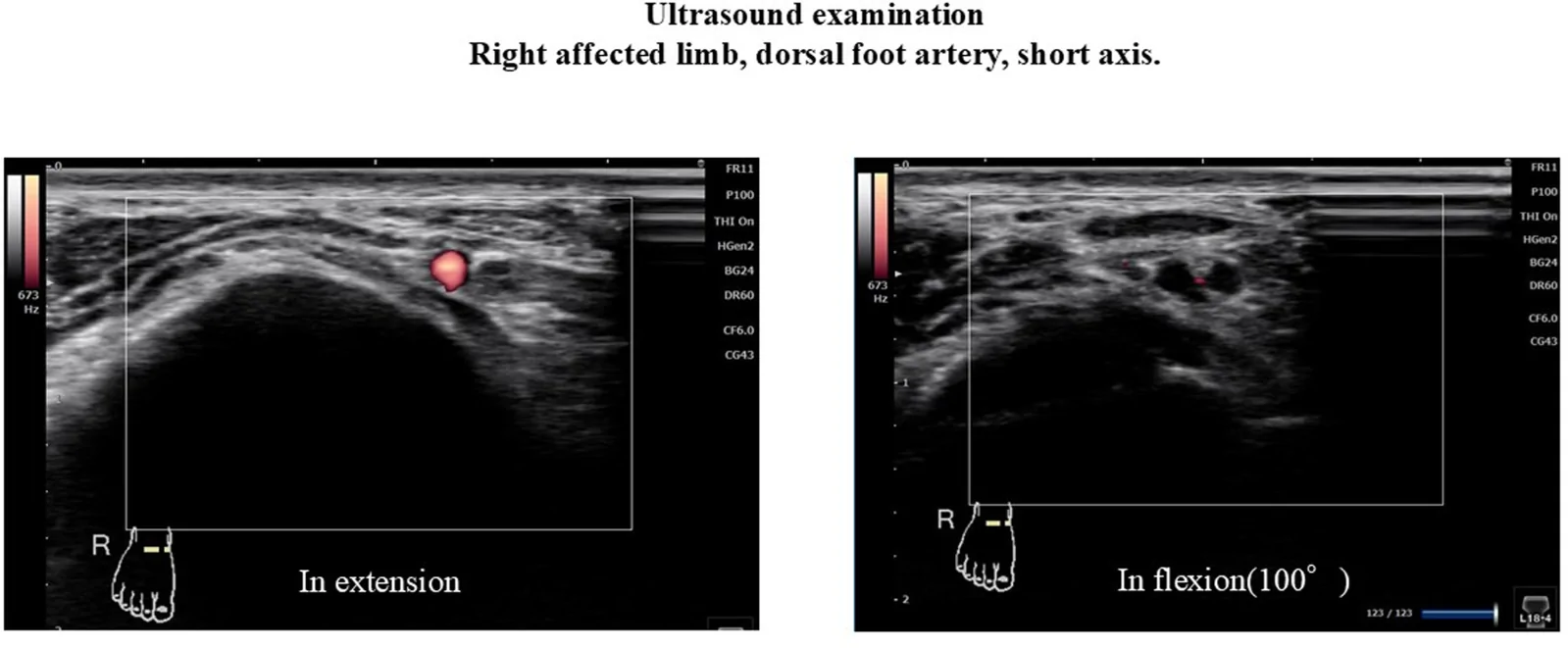
Power Doppler ultrasonography of the dorsalis pedis artery in the affected right limb (short-axis view). Blood flow is visible during knee extension but disappears during knee flexion at 100°.

## 3. Discussion

In cases of malignant soft tissue tumors of the extremities involving major vascular structures, en bloc resection with vascular reconstruction may be required to achieve local tumor control.^[1–3,10,11]^ Autologous vein grafts, such as the contralateral great saphenous vein, remain the most favorable option due to their superior patency rates, durability, and infection resistance compared with synthetic conduits.^[1,2]^ However, their biomechanical limitations – particularly reduced elasticity compared with native arteries – may contribute to functional complications when subjected to repeated or sustained joint motion.^[3]^

The present patient developed new‐onset numbness and pain specifically associated with deep knee flexion 3.5 years postoperatively. While radiation‐induced neuropathy and vasculopathy are important differential diagnoses in patients who have undergone radiotherapy,^[4–8]^ our comprehensive neurological examination and dynamic vascular assessment suggested a mechanical cause rather than radiation‐related injury. The ankle–brachial index and arterial stiffness index are established noninvasive tools for detecting peripheral arterial disease and subclinical atherosclerosis,^[12,13]^ but they may not detect position‐dependent vascular compromise.

In this case, power Doppler ultrasonography during knee flexion and extension demonstrated complete loss of flow in the reconstructed limb during flexion, while the contralateral side showed only a slight reduction. Dynamic imaging has been reported to be valuable in assessing functional vascular patency after limb‐salvage surgery,^[10,11]^ but to our knowledge, there are no previous reports directly demonstrating graft compression in this manner. This approach not only provided objective evidence for the cause of the patient’s symptoms but also facilitated patient understanding and acceptance of management strategies.

Given the growing number of long‐term survivors of limb‐salvage surgery, incorporating dynamic vascular imaging into postoperative follow‐up may be beneficial for detecting mechanical graft compromise before irreversible damage occurs. Future studies should explore graft designs or materials that combine the biocompatibility of autologous tissue with enhanced flexibility and mechanical resilience.

## 4. Conclusion

Power Doppler ultrasonography is a practical, noninvasive diagnostic tool that can detect dynamic vascular compromise, aiding in the evaluation of functional complications in patients after vascular reconstruction. Its incorporation into postoperative surveillance protocols may allow early detection and prevention of long-term disability.

## Acknowledgments

The authors used ChatGPT (GPT-5, OpenAI, San Francisco, CA, USA) and Gemini (Google DeepMind, London, UK) to assist in language editing and structural refinement of the manuscript. All content was reviewed and verified by the authors, who take full responsibility for the final version.

## Author contributions

**Conceptualization:** Taisuke Furuta.

**Data curation:** Taisuke Furuta, Tomohiko Sakuda, Yuko Nakashima, Rikuo Shinomiya.

**Investigation:** Taisuke Furuta.

**Methodology:** Taisuke Furuta.

**Resources:** Yuko Nakashima, Rikuo Shinomiya.

**Supervision:** Mitsuo Ochi, Nobuo Adachi.

**Visualization:** Rikuo Shinomiya.

**Writing – original draft:** Taisuke Furuta.

**Writing – review & editing:** Taisuke Furuta.

## References

[1] Stooker W Gök M Sipkema P. Pressure-diameter relationship in the human greater saphenous vein. Ann Thorac Surg. 2003;76:1533–8. 10.1016/s0003-4975(03)00896-8 14602282

[2] Lyzikov AA Pechenkin AA. Using femoral autovein in reconstruction of the aortoiliac segment in patients with high risk of infectious complications. Angiol Sosud Khir. 2013;19:129–32. 23531672

[3] Nishinari K Krutman M Aguiar Junior S. Surgical outcomes of vascular reconstruction in soft tissue sarcomas of the lower extremities. J Vasc Surg. 2015;62:143–9. 10.1016/j.jvs.2015.02.004 26115921

[4] Delanian S Lefaix JL Pradat PF. Radiation-induced neuropathy in cancer survivors. Radiother Oncol. 2012;105:273–82. 10.1016/j.radonc.2012.10.012 23245644

[5] Yang EH Marmagkiolis K Balanescu DV. Radiation-induced vascular disease—a state-of-the-art review. Front Cardiovasc Med. 2021;8:652761. 10.3389/fcvm.2021.652761 PMC8042773 33860001

[6] Pradat PF Delanian S. Late radiation injury to peripheral nerves. Handb Clin Neurol. 2013;115:743–58. 10.1016/B978-0-444-52902-2.00043-6 23931813

[7] Singh R Trobe JD Hayman JA Deveikis JP. Ophthalmic artery occlusion secondary to radiation-induced vasculopathy. J Neuroophthalmol. 2004;24:206–10. 10.1097/00041327-200409000-00004 15348985

[8] Kinaci-Tas B Alderliesten T Verbraak FD Rasch CRN. Radiation-induced retinopathy and optic neuropathy after radiation therapy for brain, head, and neck tumors: a systematic review. Cancers (Basel). 2023;15:1999. 10.3390/cancers15071999 PMC10093581 37046660

[9] Kerwan A Al-Jabir A Mathew G. Revised Surgical CAse REport (SCARE) guideline: an update for the age of artificial intelligence. Premier J Sci. 2025;10:100079.

[10] Emori M Hamada K Omori S. Surgery with vascular reconstruction for soft-tissue sarcomas in the inguinal region: outcomes and function. Ann Vasc Surg. 2012;26:693–9. 10.1016/j.avsg.2011.12.003 22664282

[11] Shah AC Ramanujan V Muralidharan K Raja A. Outcomes of vascular resection and reconstruction in extremity soft tissue sarcoma and bone tumors. Vasc Specialist Int. 2021;37:22–8. 10.5758/vsi.200064 PMC8021495 33795550

[12] Fowkes FG Murray GD Butcher I. Ankle brachial index combined with Framingham Risk Score to predict cardiovascular events and mortality: a meta-analysis. JAMA. 2008;300:197–208. 10.1001/jama.300.2.197 PMC2932628 18612117

[13] Alizargar J Bai CH. Value of the arterial stiffness index and ankle brachial index in subclinical atherosclerosis screening in healthy community-dwelling individuals. BMC Public Health. 2019;19:65. 10.1186/s12889-019-6398-9 PMC6332638 30646893

